# Pareto optimality, economy–effectiveness trade-offs and ion channel degeneracy: improving population modelling for single neurons

**DOI:** 10.1098/rsob.220073

**Published:** 2022-07-13

**Authors:** Peter Jedlicka, Alexander D. Bird, Hermann Cuntz

**Affiliations:** ^1^ ICAR3R - Interdisciplinary Centre for 3Rs in Animal Research, Faculty of Medicine, Justus-Liebig-University, Giessen, Germany; ^2^ Institute of Clinical Neuroanatomy, Neuroscience Center, Goethe University, Frankfurt/Main, Germany; ^3^ Frankfurt Institute for Advanced Studies, Frankfurt am Main, Germany; ^4^ Ernst Strüngmann Institute (ESI) for Neuroscience in Cooperation with Max Planck Society, Frankfurt am Main, Germany

**Keywords:** Pareto front, energy efficiency, multi-objective optimization, parameter space, performance space, ion channel correlations

## Abstract

Neurons encounter unavoidable evolutionary trade-offs between multiple tasks. They must consume as little energy as possible while effectively fulfilling their functions. Cells displaying the best performance for such multi-task trade-offs are said to be Pareto optimal, with their ion channel configurations underpinning their functionality. Ion channel degeneracy, however, implies that multiple ion channel configurations can lead to functionally similar behaviour. Therefore, instead of a single model, neuroscientists often use populations of models with distinct combinations of ionic conductances. This approach is called population (database or ensemble) modelling. It remains unclear, which ion channel parameters in the vast population of functional models are more likely to be found in the brain. Here we argue that Pareto optimality can serve as a guiding principle for addressing this issue by helping to identify the subpopulations of conductance-based models that perform best for the trade-off between economy and functionality. In this way, the high-dimensional parameter space of neuronal models might be reduced to geometrically simple low-dimensional manifolds, potentially explaining experimentally observed ion channel correlations. Conversely, Pareto inference might also help deduce neuronal functions from high-dimensional Patch-seq data. In summary, Pareto optimality is a promising framework for improving population modelling of neurons and their circuits.

## Graphical abstract

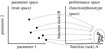

## 1. Ion channel degeneracy in population models of neurons

**Figure 1.**
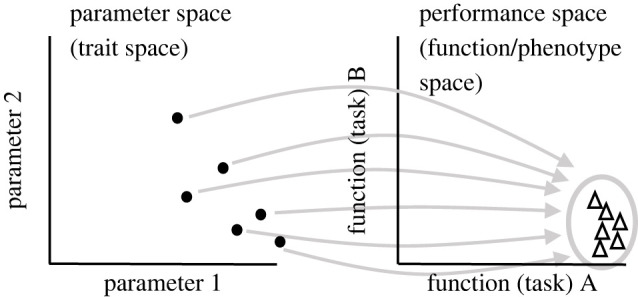
Degeneracy in the parameter space of biological systems (e.g. neurons with ion channels). Multiple disparate parameter configurations in the parameter (trait) space (e.g. ion conductance space) can lead to similar functional phenotypes optimized for a given task A (e.g. dendritic computation). In degenerate systems such as our brain, there is a multiple-to-one mapping between the parameter space and the phenotype space at all scales including the scale of ion channels and nerve cells (and their circuits). Each point (triangle) may represent a single neuron in a multidimensional parameter (performance) space. The schematic shows a 2D space but in real systems, parameter and performance space can have different numbers of dimensions (see also figure 8). The degeneracy and Pareto optimality concepts can be applied to any number of dimensions. For three-dimensional version of a similar schematic see, for example, fig. 4 in Mishra & Narayanan [8]. Throughout this article, we consider ‘parameter space’ and ‘trait space’ to be synonyms. Similarly, we consider ‘performance space’, ‘functional space’, ‘phenotype space’ and ‘output space’ to be synonyms. This applies also to (neuronal) ‘tasks’, ‘objectives’ and ‘functions’.

Landmark studies have shown that multiple different parameters of ion channels can generate similar activity both at the level of single cells [1] as well as neural circuits [2,3]. This multiple-to-one mapping between combinations of ion channel parameters and cell or circuit phenotypes has been termed ion channel degeneracy [4] or non-uniqueness [5,6]. Degeneracy [7] is present at all scales of the brain (figure 1). Its importance for the flexibility and robustness of brain functions has been increasingly acknowledged in recent years (for reviews see [9–11]). Accordingly, ion channel degeneracy has been linked to the flexibility [4] and robustness of neuronal behaviour [10,12,13].

Several groups have adopted computational insights and methods of the above-mentioned landmark studies [1,2] to explore ion channel degeneracy in different types of neurons (e.g. [5,14–23]). This approach has been successfully used even outside of neuroscience, for example in heart cell physiology [24]. It has been called population- [23–25] or database- [16,17] or ensemble-modelling [18]. Population-based computer models have provided a better understanding of cell-to-cell as well as animal-to-animal variability of electrophysiological and ion channel expression data [3,25,26]. Instead of a ‘one-size-fits-all’ approach in which a computer model simulates average properties of a nerve cell or heart muscle cell, the population-based approach constructs and validates large populations of realistic cellular models that differ in their ion channel configurations and reflect the variability of experimental data [24,27]. Recent work has also shown that such population models may allow pharmacological predictions *in silico*, thus complementing, and partially replacing animal experiments [23,28].

Ion channel degeneracy applies not only to intrinsic cellular properties but also to extrinsic synaptic properties [2]. Already the first landmark studies have shown that many disparate configurations of synaptic and intrinsic conductances are able to generate similar neuronal behaviour as well as similar (functional) network behaviour [2] (see also [29–33]). Therefore, although in this article we focus on degeneracy of intrinsic ion channels at the cellular level, the concepts of degeneracy and Pareto optimality [34] can be extended also to extrinsic synaptic channels and to the level of neuronal circuits.

## 2. The problem of the large and complex parameter space of functional models

Given the importance and the success of the population modelling approach, it would be desirable to further improve its predictive power. This would facilitate clinically relevant predictions about the role of ion channels in neurological diseases with known ion channel expression correlates, such as epilepsy. However, to achieve this, we need to find a solution to one particularly problematic issue of population modelling. Due to ion channel degeneracy, one can obtain similar neuronal computational, functional and electrophysiological properties with widely different parameter combinations in any given neuronal biophysical model. The problematic issue is that it is unclear which compositions of ion channels and their parameters, all of which generate realistic (functional) electrophysiological behaviour, are in reality preferred by evolutionary selection. In other words, it is not understood, which ion channel configurations in a vast population of valid models are more likely to be found in the brain. We know that often there is a degenerate multiple-to-one mapping between parameter (or trait) space of ion channels and phenotype (or performance/function) space of neurons (figure 1). However, we lack a theoretical framework to fully constrain this mapping in a biologically realistic manner.

Thus, it is an unresolved question whether naturally occurring configurations of neuronal parameters occupy a large or a restricted subspace in the large, theoretically possible parameter space. The complex shape of the valid parameter space has been explored before (e.g. [15,35,36]). However, there is a need for universal guiding principles that would further constrain the shape of the parameter space to those models that most likely represent real neurons found in nature. Such a principle would help address the following questions. Are naturally occurring instances of real neurons (and their circuits) confined to low-dimensional manifolds or rather scattered widely over the entire parameter space [37]? In case a neuron type has *n* ion channel parameters, each instance of the neuron can be represented as a point in an *n*-dimensional parameter space. Can complex *n*-dimensional conductance spaces [35] be reduced to low-dimensional subspaces? If real (naturally occurring) configurations of parameters were restricted to low-dimensional manifolds [8], it would greatly enhance our understanding of neuronal systems. It would potentially allow us to infer most unknown parameters from a small subset of known parameters [37].

Intriguingly, 10 years ago, in their pioneering research, computational systems biologists started using Pareto optimality to show that evolution selects phenotypes that are located in low-dimensional manifolds (e.g. lines, triangles) of parameter space [38]. Pareto theory predicts that such a low-dimensional geometry of the parameter space would be found in nature. The framework of Pareto optimality explains it as a consequence of evolutionary optimization of the phenotypes for their multiple tasks (functions) and making optimal trade-offs between the tasks [37,38] (for a recent neuroscience review see [34]).

## 3. Multi-objective Pareto optimality as a geometrically elegant solution for simplifying parameter space

**Figure 2.**
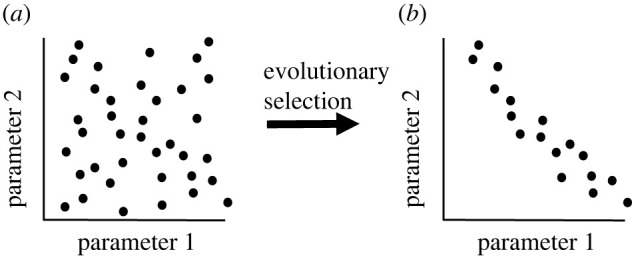
Evolutionary selection based on trade-offs between multiple tasks can remove suboptimal points from (ion channel) parameter space. Each (neuronal) phenotype can be seen as a point in a two- or *n*-dimensional parameter space. There are two possibilities for the geometry of parameter space, as follows. (*a*) The (ion conductance) parameters that contribute to the function(s) of a neuron fill the entire parameter space. (*b*) Parameters occurring in nature (in real neurons) are restricted to a small subspace (or a curve) of the parameter space because evolution removes inefficient or ineffective parameter configurations. This can be generalized to any number of dimensions. Evolution can confine a high-dimensional parameter space to a low-dimensional manifold [37].

Evolutionary restriction of a complex parameter space to a simpler subspace or a low-dimensional manifold is *per se* a plausible and realistic assumption (figure 2). However, it remains unclear what additional principle can help us in practice to reduce the parameter space of degenerate ion channels in populations of neuron models. Ideally, such a principle would allow us to identify or at least approximate the shape of the subspaces or manifolds selected by evolution. Pareto optimality linked to evolutionary trade-offs [39] is a promising candidate for such a general and at the same time a practical principle.

Usually a neuron (in fact any artificial or a biological system) has to fulfil more than one task at the same time. For example, it must generate functional electrical behaviour (e.g. dendritic spikes and/or somatic bursting) and/or maintain its stable (fast or slow) firing and at the same time expend as little energy as possible. In addition, a neuron often has to be robust against perturbations and/or flexible enough to respond to a wide input range. Nevertheless, typically its performance cannot be optimal for all of these separate tasks. Thus, neurons (and their circuits) face a fundamental optimization problem of finding an optimal trade-off among multiple objectives [40,41]. If there is a competition between multiple tasks, then evolutionary multi-task optimization can lead to a ‘tug-of-war’ dynamics [42] pulling neurons towards an equilibrium with Pareto optimal multi-task solutions.

**Figure 3.**
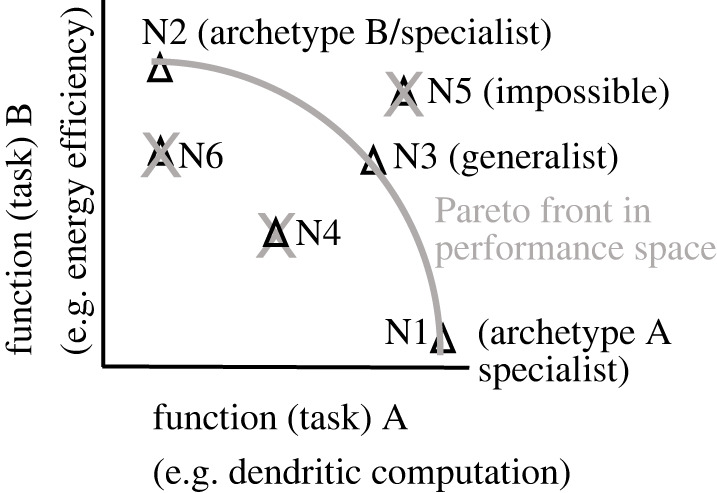
Neuronal phenotypes that cannot be outperformed in all tasks simultaneously are Pareto optimal and lie on the Pareto front. Neuronal phenotypes, which correspond to different ion channel configurations, can be plotted in performance space based on their performance in 2 (or *n*) tasks. N1, N2 and N3 neurons outperform N4 and N6 in at least one task. Best phenotypes for a given task (A or B) are referred to as archetypes (A or B, respectively). Pareto optimal neurons that are close to archetypes are called task specialists. Neurons that are in the middle can be called generalists. Based on Alon [39]. See also Pallasdies *et al*. [34].

**Figure 4.**
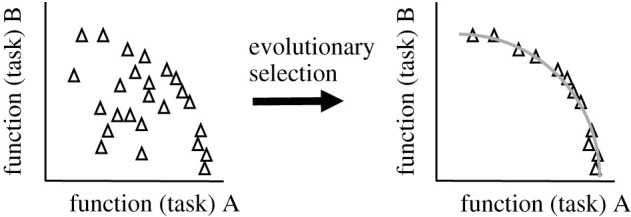
Evolutionary trade-offs between different tasks reduce the performance space to a Pareto front. The key hypothesis is that evolutionary selection based on multi-task trade-offs removed suboptimal neuronal phenotypes from performance space and greatly simplified performance (and the corresponding parameter) space. Based on Alon [39]. Caveat: if measured neuronal phenotypes do not lie on a Pareto front predicted by a Pareto analysis, this could mean that the analysis neglected some important tasks or that neurons are not Pareto optimal for the studied tasks (see [34]).

**Figure 5.**
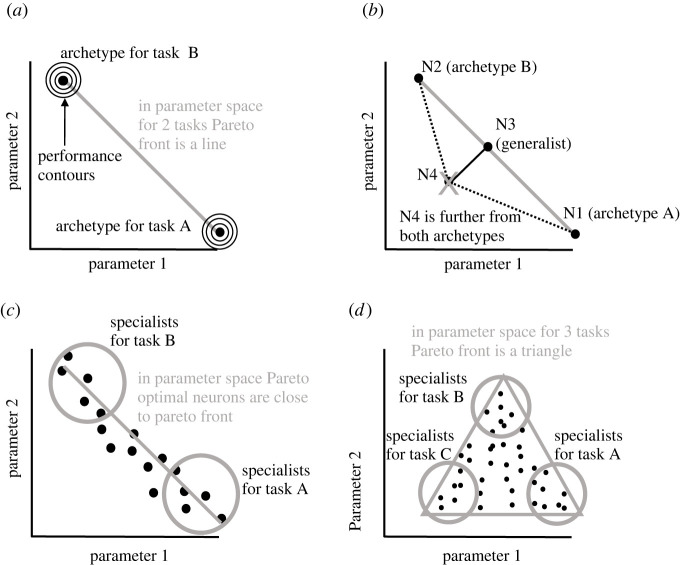
Trade-offs between 2 or 3 tasks reduce parameter space to low-dimensional Pareto fronts in the form of a line or a triangle, respectively. (*a*) Archetypes are located at peaks of a task performance. Contours indicate a monotonic drop in performance for locations further away from archetypes. (*b*) Nonoptimal neurons (N4) are more distant from archetypes than neurons on the Pareto front (N1,2,3), which is the line between archetypes (N1,2). (*c*,*d*) Hypothetical neurons that are concurrently optimized for 2 or 3 objectives (e.g. dendritic computation, its energy efficiency and robustness) would be found on a line segment or inside a triangle with specialists near the 2 or 3 vertices (archetypes), respectively. Based on Alon [39].

A key evolutionary hypothesis is that if multiple competing tasks affect the fitness of a phenotype then evolution will select individual phenotypes with optimal performance for, potentially different, combinations of those tasks (for trade-offs between them, see [39]). In such a case, Pareto optimality may help identify the sets of neurons, for which evolution solved the multi-task optimization problem. By definition, the performance of such a set of Pareto optimal neurons cannot be improved for any task without decreasing their performance for some other task. This means that no other plausible neuron can dominate a Pareto optimal neuron by outperforming it at all tasks simultaneously. The set of Pareto optimal neurons form a so-called Pareto front in task or function space. By contrast to the parameter space, the space of plausible neuronal functionality is typically restricted, with the Pareto front forming part of the boundary manifold between plausible and implausible regions. A neuron belongs to a Pareto front if and only if the following condition is satisfied [43]: for any other distinct neuron in the population, there must exist at least one task at which the Pareto front neuron is strictly better. We can examine such Pareto front sets of neurons first in performance (figures 3 and 4) and then in parameter space (figure 5).

Let us assume that neurons need to optimize their biophysical design (ion channel parameters) for two tasks simultaneously, for example low energy expenditure and dendritic computation. If one neuron (e.g. N4 in figure 3) underperforms another neuron (e.g. N3) at both tasks simultaneously then it does not belong to the Pareto front and has been likely eliminated by evolutionary selection. If we remove all neurons that are outperformed (i.e. dominated) by other neurons, we get the Pareto front. Thus, the set of neurons that cannot be outperformed concurrently at both tasks (objectives) is the Pareto front set. The neurons (phenotypes) that achieve peak performance for one objective (N1 and N2) are called archetypes [38,39]. Archetypes are phenotypes with best combinations of parameters (traits) for given tasks. N1 is the neuronal phenotype with such a combination of ion channel parameters that leads to the best performance in dendritic computation. By contrast, N2's ion channel parameters support its best performance in energy efficiency (i.e. economy: defined e.g. as low ATP consumption per spike, see below). N5 represents an impossible configuration of parameters with effective dendritic computation, but which is not achievable at such a high energy efficiency.

Strikingly, landmark computational studies have shown that the Pareto optimality principle can elegantly simplify the geometry of the parameter space, in fact more clearly than it can the geometry of the performance space [38,44]. Two tasks or objectives push neurons with an optimal trade-off to a low-dimensional subregion in parameter space that corresponds to a Pareto front. Interestingly, irrespective of the number of parameters (traits), in the case of two tasks, the Pareto front must have a shape of a line connecting two archetypes (figure 5*a*). The reason is that any neuron that is not located on the line (e.g. N4 in figure 5*b*) is necessarily further away from the two archetypes (N1, N2) than any neuron on the line (e.g. N3). This is the case in parameter space, but not in performance space. The performance landscape can be visualized in the parameter space by drawing performance contours (figure 5*a*) around archetypes [38]. Archetypes represent peaks of performance for single tasks. Importantly, performance decreases with a growing distance from archetypes (in parameter space). For geometrical reasons, each neuron belonging to the Pareto front must have a lower total distance to both archetypes than any neuron outside the Pareto front. The lower distance to both archetypes means higher simultaneous performance in both tasks. Pareto optimal neurons that are near the ends of the Pareto line segment are specialists for one of the two tasks, while those in between are generalists for both tasks.

Similar geometrical reasoning can show that, for three tasks, the Pareto front must have the shape of a triangle in parameter space (figure 5*d*). Intriguingly, this is again true irrespective of the number of dimensions of the parameter space. Again, whereas specialists, optimal for a single task, concentrate close to one of the three corners (archetypes), generalists, optimal for combinations of three tasks, occupy the region in the middle. This can be extrapolated to any number of tasks. For four tasks, Pareto front is a tetrahedron with four corners or vertices (archetypes). For *n* tasks, the Pareto front is a polytope with *n* vertices or corners [39,44].

Importantly, these geometrical insights hold under three assumptions about task performance [38,44]: (1) performance decays monotonically with increasing remoteness from the peak (archetype); (2) there is one point representing a global peak; (3) all performances decay with the same metric distance from corresponding peaks. However, even after violating these conditions, approximate Pareto fronts with relatively simple shapes can still emerge, still having archetypes as vertices. The vertices are then connected by mildly curved instead of straight lines, still corresponding to where the performance contours for different tasks lie tangentially to one another [38,44].

## 4. Evolutionary trade-offs between functionality (effectiveness) and energy efficiency (economy) of neurons

How could we take advantage of the above geometrical principles [38,39] derived from Pareto optimality? How can we apply them to reduce the parameter space of degenerate neuronal models? Ion channel degeneracy implies multiple valid solutions (i.e. a population of solutions) for realistic voltage traces. The valid solutions can fill large and distributed regions of parameter space (see e.g. [15,35,36]). However, although being valid with respect to reproducing voltage traces, individual models in a population differ regarding their optimality for additional functions or objectives. One such important additional objective is energy efficiency (i.e. economy or low ATP expenditure; cf. [34]).

It is well established that to achieve their computational goals, brain circuits and nerve cells consume large amounts of energy [45–47]. Therefore, their anatomical and physiological properties are likely to be optimized for a fundamental trade-off between function-effectiveness (i.e. effectiveness) and energy efficiency (i.e. economy). This would be in close agreement with the Pareto optimality concept although in most neuroscience studies it has not been named as such (for a recent general review, see [34]). It has been increasingly recognized that evolution has optimized neurons and their circuits for best simultaneous performance in terms of functional effectiveness and economy [34,40,48]. Thus, Pareto optimality for the trade-off between effectiveness and economy, which has been used to better understand non-neuronal systems [37] can also be applied explicitly to neurons as a general principle. In line with this, in neural information theory, accumulating computational and experimental evidence shows that neurons are not optimized for processing maximum amounts of information but rather maximum amounts of information per energy cost [49–54]. Although not explicitly using Pareto theory, several studies have indicated that both extrinsic (synaptic) as well as intrinsic ion channel properties of neurons are concurrently optimal for high energy efficiency (economy) and effective information processing and its biophysical implementation (e.g. [55–64]).

**Figure 6.**
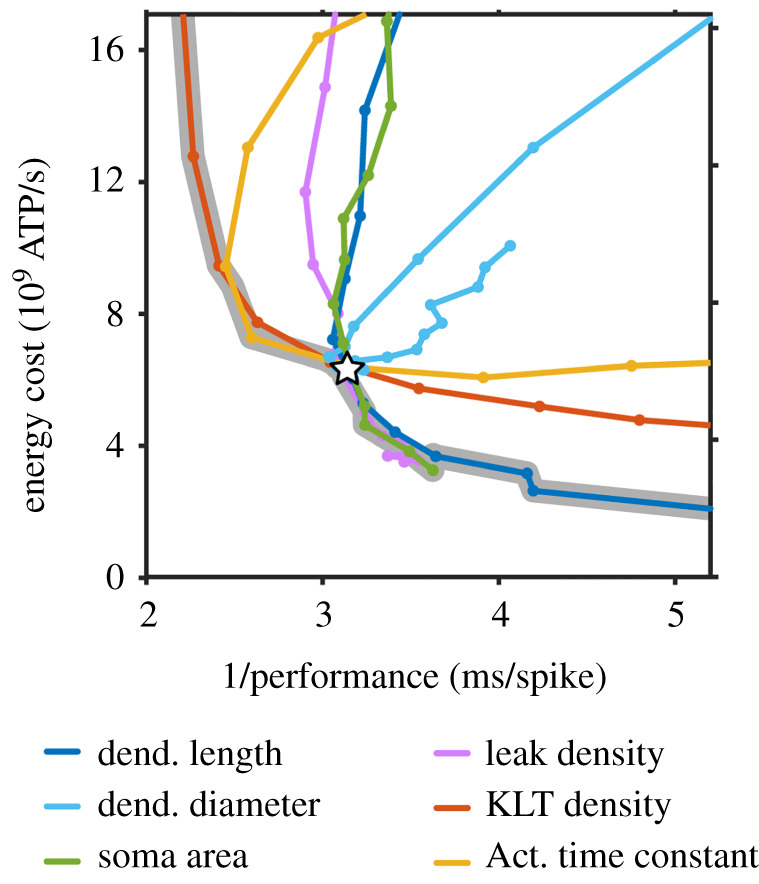
Simulated neurons in the medial superior olive (MSO) are Pareto optimal for a 2-task trade-off between functional effectiveness and energy efficiency (economy). The figure shows a performance space for 2 tasks simulated in MSO neuron models. Simulations were run in conductance-based neuronal models with simplified morphologies. Performance in dendritic computation (task A, *x*-axis) in the form of temporal coincidence detection for inputs (underlying sound localization in the MSO) is plotted against performance with respect to energy efficiency (task B, *y*-axis). The grey line indicates the Pareto front with models that are optimal for the trade-off between the 2 tasks. Coloured curves show models with varied morphological and biophysical (ion channel) parameters. Energy cost was estimated using a standard approach for converting ionic currents into ATP. KLT: low threshold potassium current. Note that a model, which was well constrained by experimental data (open star), is close to the Pareto front and displays strong performance in coincidence detection and high energy efficiency (i.e. low energy cost). Reproduced from Remme *et al*. [65], licensed under Creative Commons Attribution License. https://doi.org/10.1371/journal.pcbi.1006612.g004.

Intriguingly, a recent study used conductance-based modelling to show explicitly that neurons in the medial superior olive (MSO) are close to the Pareto front set of models in performance space [65]. The Pareto front neurons were optimal for a two-task trade-off between energy expenditure and a well-known MSO neuronal function, namely detection of temporal coincidence in input signals (figure 6). The temporal coincidence detection is crucial for the computation of the direction of sound source in the MSO. Remarkably, a default model (open star in figure 6) that was experimentally well constrained for biophysical (ion channel) and morphological parameters exhibited high energy efficiency and at the same time high functional (computational) effectiveness. This pioneering study is probably the first publication that has explicitly applied the Pareto optimality theory to an effectiveness-economy trade-off in a conductance-based neuronal model (figure 6). The modelling work has indicated that neurons minimize their energy costs related to their ion channel parameters as long as their computational function remains intact [65].

Interestingly, the morphological parameter space of neuronal dendrites and axons can be understood to some extent separately from the biophysical parameter space [66,67]. A morphological modelling study by Cuntz *et al*. [68] showed that dendritic trees of neurons search for a compromise between two costs: cable length and conduction time. Minimization of cable length can be seen as minimization of energy cost (i.e. maximization of efficient wiring), whereas minimization of conduction time can be seen as effective signal propagation [43,69,70] (see also fig. 1 in [34]). A more recent publication has confirmed and extended findings of Cuntz *et al*. [68,70] showing that by implementing optimal solutions for the trade-off between cable length and conduction time, dendritic trees seem to achieve Pareto optimality [71]. As a note of caution, this conclusion should be corroborated by further explorations, using a more extensive collection of random tree models as a negative control.

Thus, although many open questions about the relationship between morphology and biophysics in neuronal models remain (e.g. [72]), these studies indicate that dendrite morphology is well constrained by optimal wiring alone (if defined as optimal dendritic structure for minimizing the cable length and the conduction time [68,70]). Similar observations have been made for axonal connections [43,73–76] (but see also [77]). Therefore, in this article we focus mostly on the conductance space and do not discuss the morphological space of population neuronal models and its impact on the variability and robustness of electrophysiological behaviour (for this topic see e.g. [78,79]) or their potential interactions.

## 5. Effectiveness-economy trade-offs may simplify conductance space of population models of neurons

So far the Pareto optimality theory with a focus on effectiveness-economy trade-offs has not yet been explicitly used to address directly the problem of ion channel degeneracy in population models of neurons (but see also [5], and the discussion below). We argue that multi-objective Pareto optimality, which can be estimated for trade-offs between known (but to some extent also unknown, see Pareto task inference below) computational functions and energy efficiency could be a very fruitful theoretical framework for improving population models of neurons. Energy costs of individual conductance-based models in populations of valid single- or multi-compartmental models are usually not considered. However, an estimation of energy costs for a given ion channel configuration is relatively straightforward and easy to implement (see e.g. [55,65]). Currents flowing through ion channels in compartmental models can be collected and converted to ATP costs. The estimated ATP amount is proportional to energy consumed by ATP-driven pumps, which maintain transmembrane concentration gradients of sodium, potassium and calcium ions. ATP calculated in this way is a good approximation for the energy costs of conductance-based models [45,65]. Therefore, in the context of the Pareto optimality framework, we suggest that, whenever possible, energy efficiency should be added as an additional objective to inform the search for most realistic configurations of ion channel parameters. Moreover, we encourage modellers to extend the objectives by evaluating the models not only by their voltage trace features but also by their performance in well-defined computational functions. Examples would include for instance coincidence detection of MSO neurons [65] coincidence detection in the form of BAC firing of cortical layer 5 pyramidal neurons [80,81], orientation tuning of layer 2 pyramidal neurons [82] spatial tuning of grid cells [83] and place cells [84], pattern separation in dentate granule cells [85], or motion detection in direction-selective T4 neurons [86].

It is important to note that feature-based multi-objective optimization has already been established before as an extremely helpful method for tuning compartmental models [5]. In their landmark study, the authors used genetic algorithms to optimize multiple objectives in the form of selected features of experimental voltage traces such as frequency, timing or width of action potentials [5]. This approach has been very successful as a feature-based optimization tool [5,19,20,22,81,87–89]. Here we argue for an extrapolation of multi-objective optimization from spiking features to additional neuronal functions and their energy costs. This would extend population neuronal modelling beyond reproducing electrophysiological features toward capturing evolutionary trade-offs between physiological functions and their energy costs. In other words, conceptually we suggest using multi-objective trade-offs in a more general evolutionary context (cf. [90]). Furthermore, we propose using the above-described geometrical principles [38,39] for exploring whether it is possible to reduce high-dimensional parameter spaces of ion conductances to low-dimensional manifolds. Importantly, multi-task Pareto optimization or selection of neuronal models based on function-economy trade-offs can be combined with or complement standard multi-objective optimization based on trade-offs between voltage features. The hope is that such a complementary use of the two non-exclusive overlapping approaches [5,65] can further constrain the parameter space in a biologically realistic manner.

Indeed, recent modelling efforts suggest that such an extended multi-objective optimality framework could be a promising approach for tackling ion channel degeneracy in populations of conductance-based models. New computational studies employing population models of neurons or neuronal circuits have provided further hints that effectiveness-economy trade-offs contribute to a better understanding of the complex parameter space and its degeneracy. One study [91] used, in a first step, the classical feature-based multi-objective optimization [5,81] to generate multi-compartmental population models for layer 5 pyramidal tract neurons (L5 PCs). Then, in a second step, the feature-optimized models were further analysed for their energy efficiency using the above-mentioned ATP estimation approach based on monitoring ionic currents [65]. Not surprisingly, the population models of L5 PCs showed extensive degeneracy of ion channels since nonlinear dendritic computation emerged from a large range of ion channel configurations. Notably, their computational analysis identified models that were efficient in terms of energy as well as effective in terms of dendritic computation. The nonlinear dendritic computation was assessed based on BAC firing [80,92] and dendritic calcium spikes, which are thought to be important for conscious perception [93–95].

**Figure 7.**
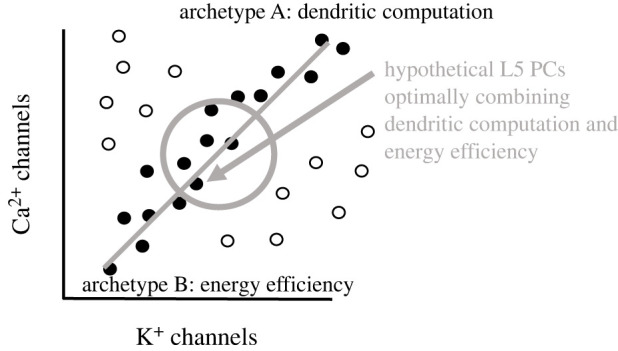
L5 pyramidal tract neurons (L5 PCs) might be Pareto optimal for a 2-task trade-off between dendritic computation and energy efficiency. Inspired by population modelling of L5 PCs by Bast & Oberlaender [91], we hypothesize that Pareto optimal parameter space (i.e. the Pareto front) for K^+^ and Ca^2+^ channels (referring mainly to fast noninactivating potassium channel Kv3.1 and high-/low-voltage activated calcium channels) might be a line. L5 PC models with low Kv3.1 and Ca^2+^ channel expression (in the dendritic hot zone) compromise optimally between dendritic computation (i.e. dendritic spikes and BAC firing) and low energy costs [91]. Grey line: a hypothetical Pareto front with models jointly optimal for combined 2 tasks (filled circles). Grey arrow: a hypothetical model with a relatively low (experimentally observed) expression of Kv3.1 is close to the Pareto front and displays a good combined performance in energy efficiency (archetype 1) and dendritic computation (archetype 2). Open circles: Pareto non-optimal models. See text for more details.

Curiously, the L5 PC models with energy-efficient dendritic computation displayed a low expression of fast non-inactivating potassium channels and high-voltage activated calcium channels in the dendritic calcium hot zone [91], which corresponds to a major site of dendritic spike generation. Consistent with the idea that evolution selects energy-efficient neuronal phenotypes [96], low expression of potassium channels in distal apical dendrite has been observed before in real neurons [97]. Although the authors did not perform Pareto analysis for the economy-computation trade-off, it is tempting to speculate that the models with a best compromise for the two tasks (dendritic computation and energy efficiency) would lie close to the Pareto front in parameter space for ion channels (figure 7). Future experimental and computational analyses might reveal whether the Pareto front for optimal ion channels in L5 PCs resembles a line segment connecting the two archetypes of dendritic computation and energy efficiency (or a triangle in case of three tasks, etc.). Indeed, the authors concluded that L5 PCs do not exploit all possible parameter combinations but ‘select those optimized for energy-efficient active dendritic computations'. Interestingly, morphological variability did not seem to play a major role, suggesting that dendritic structure is constrained mainly by optimal wiring (as we mentioned before) and does not greatly affect ion channel parameters.

Another recent study [32] explored how energy efficiency and temperature robustness affect parameter space of population models for the canonical circuit of the crab stomatogastric ganglion, in which parameter degeneracy was discovered for the first time [2]. The authors combined population modeling with a new machine learning method for estimating parameters of mechanistic models [98]. As expected, energy efficiency reduced the parameter space for realistically behaving models [32]. However, the remaining parameter space was still large and degenerate so that disparate parameter combinations still led to well performing models in terms of energy efficiency and network behaviour. In addition, somewhat surprisingly, increased robustness to temperature did not always cause increased energy consumption. This suggests that in this circuit there might not be a significant trade-off between energy cost and robustness to alterations in temperature. Nevertheless, it does not exclude the possibility that future research employing Pareto optimality theory will discover other tasks (e.g. robustness to other, temperature-unrelated perturbations) that would further constrain parameter space and find stronger trade-offs with energy efficiency. Notably, although the authors did not study Pareto fronts in conductance space, their simulations predict that sodium and calcium conductances contribute significantly to energy costs and are therefore ‘less variable in nature than expected by computational models only matching network activity’. This is in agreement with the hypothesis that evolution does not implement all possible parameter combinations but selects their subsets (see figures 2 and 4). Interestingly, the work showed also that individual neuron models could be tuned for low energy costs independently from network activity and then used to construct energy-efficient circuit models. This strengthens the idea that Pareto analysis of economy-function trade-offs can be applied not only to circuits but also to single-cell models of neurons.

## 6. Pareto inference for deducing neuronal functions from high-dimensional patch-seq data

Until now we have described possible applications of Pareto theory to known tasks (or functions and their energy costs) of neurons. However, the tasks of most neurons and neural circuits are still not fully understood. Moreover, even if we knew the functions, we might not be able to estimate the associated performance in performance space. Surprisingly, even if no (or not all) functions and corresponding performances of neurons are known, the framework of Pareto optimality can still be used and provide interesting insights.

**Figure 8.**
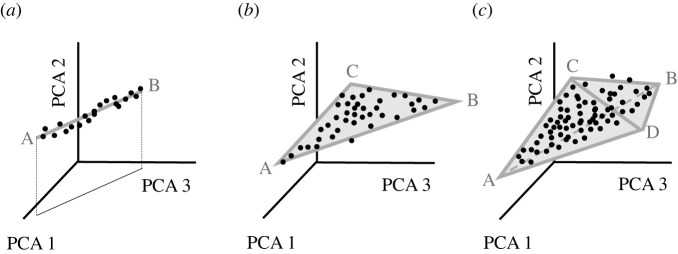
Pareto task inference (ParTI) may help infer main functions of neurons from single-cell ion channel expression data. High-dimensional datasets, e.g. ion channel expression data from Patch-seq experiments, can be reduced to a 3-dimensional parameter space by principal component analysis (PCA). According to Pareto theory, trade-offs between n tasks will lead to data points filling out geometrical objects (Pareto fronts) with a shape of polytopes with *n* vertices (2 in A, 3 in B, 4 in C). The vertices (sharp corners) in measured datasets could be used to infer the key tasks of given neuron types as shown previously for nonneuronal cell types (e.g. liver cells—see [100]). However, future research is needed to clarify whether doing PCA first would give the most significant functional archetypes or would lead to a loss in archetypes by projecting to a lower-dimensional space. The figure is based on Alon [39].

As mentioned above, in parameter space (but not in performance space), evolutionary multi-task optimization leads to Pareto fronts with specific geometrical shapes (see figure 5 for a 2D parameter space). A trade-off between two, three, four or *n* tasks leads to a Pareto front shaped as a line segment, a triangle, a tetrahedron or a polytope with *n* vertices and an (*n* – 1)-dimensional surface [38,39,44,99]. Optimal neuronal phenotypes would be expected to lie inside such polytopes whose vertices represent the archetypes for each task. Remarkably, the theory [39] predicts that Pareto fronts with polytope shapes and sharp vertices will emerge in parameter space independently from the number of measured parameters (i.e. the number of dimensions; see figure 8 for a three-dimensional parameter space). For example in two-, three- or higher-dimensional parameter space, two tasks always lead to a one-dimensional line segment (a curve) as the corresponding Pareto front. The reason for this is that the projection of the line segment to a plane is again a line segment ([39]; figure 8*a*). Therefore, in theory, these geometrical shapes (especially their vertices) should be identifiable in experimental datasets irrespective of which or how many parameters were measured [39]. Conveniently, if biological data can be fit to the polytopes (i.e. lines, triangles, tetrahedrons etc.) then the sharp vertices or corners can be exploited to infer biological tasks from experimental data (figure 8). This innovative approach has been termed Pareto task inference (ParTI, [38,99]).

ParTI has been successfully applied to diverse datasets including morphologies and life history traits in animals [101,102] or parameters in biological homeostatic systems [37] as well as gene expression in bacteria [38], liver cells [100] and cancer cells [90,103]. Motivated by the success of ParTI even for single-cell data, we believe that ParTI applied to Patch-seq data (table 1 in [104]) might provide new insights into neuronal functions. Patch-seq experiments generate large amounts of multimodal and high-dimensional data for ion channel expression, electrophysiological behaviour and dendritic morphologies of neurons (e.g. [105–108]). Unfortunately, higher than three-dimensional datasets are difficult to visualize. However, Pareto optimality makes it plausible that neuronal tasks (such as for example nonlinear dendritic computations, sparse or fast firing, etc. and most likely always also energy efficiency) simplify the geometrical shape of experimentally observed parameter space of Patch-seq datasets to low-dimensional manifolds. Typically, Patch-seq datasets are analysed by common data clustering analyses. However, clustering analysis presupposes that neuronal data is structured in separate clusters [39] although ion channel degeneracy may lead to data continuity [109] (see also [36,91]). Accordingly, ParTI may be more suitable for a high-dimensional continuum of Patch-seq data (e.g. [109]) than clustering analysis [39]. High-dimensionality of datasets is not a great problem since the Pareto optimal geometrical shapes should emerge irrespective of dimensionality. Besides, dimension reduction methods (e.g. principal component analysis, PCA) can help visualize projections of the presumably Pareto optimal shapes in two-dimensional or three-dimensional parameter space (figure 8). Interestingly, the above-mentioned population modelling of L5 PCs [91] discovered a highly significant correlation of energy efficiency with the first principal component (PCA1) in hybrid function/parameter space for ion channels (space consisted of total charges flowing through ion channels). Together with non-neuronal cellular examples [39,100], this suggests that ParTI (or its improved version [110]) might be able to reveal biologically important tasks of analysed neurons when applied to a high-dimensional ion channel space reduced to three dimensions by PCA.

PCA on a high-dimensional dataset might mask some archetypes by projecting any true high-dimensional polytopes onto a lower-dimensional space. In this case, archetypes might become unidentifiable as their corresponding vertex is projected to the interior of the new polytope. However, it is also possible that by increasing variability along certain dimensions in PCA trait space, while reducing total dimensionality, significant archetypes would be easier to identify in potentially noisy data (figure 8). Further research is needed to categorize when exactly each situation will apply, but PCA will remain a useful tool alongside ParTI when used appropriately.

Aside from that, ParTI is based on statistical inference of Pareto optimality [99] comparing the set of solutions to randomly shuffled data under the assumption that phenotypic traits are independent and the data are uncorrelated. It has been pointed out that this may not be the case in biological datasets due to phylogenetic correlations of traits [111,112] (see also [34]). This would make the identification of Pareto fronts in high-dimensional datasets prone to errors. Nevertheless, a recent study has addressed these problems by introducing a new algorithm accounting for the phylogenetic dependence of traits [110]. In any case, it will be interesting to test whether carefully applied ParTI can infer known (or also unknown) functions of neurons from the geometrical shapes of Patch-seq datasets and their sharp corners in parameter space.

Del Giudice & Crespi [41] have described basic functional trade-offs between four universal tasks of neural systems, namely (1) functional performance (termed ‘efficiency’ in their article, synonymous with ‘effectiveness’ in our article), (2) energy efficiency (synonymous with ‘economy’), (3) robustness and (4) flexibility (see their article for a concise definition of these four universal properties). Converging evidence indicates that trade-offs between the four tasks profoundly shape cognitive, neuronal and synaptic phenotypes [41]. Correspondingly, it is an exciting question whether these basic four functional properties (or their derivatives) and their trade-offs can be identified, disentangled and clarified with the help of ParTI-analysis of experimental data. Future ParTI-based neuroscience studies might focus on inferring brain region-specific, cell type-specific or/and universal tasks of neurons across brain regions (cf. [90]).

## 7. Open questions

Multi-task Pareto optimality is a promising but still largely unexplored framework for studying ion conductance space of neurons and their models. For example, it remains an open question whether Pareto analysis will show that real neurons with their naturally occurring ion channel parameters lie on modelling-based Pareto fronts or not. If data showed that neurons were distant from a predicted Pareto front this could mean that the Pareto analysis did not include the important (i.e. evolutionarily relevant) tasks or that neurons were not close to being Pareto-optimal [34]. In any case, the Pareto framework will provide new testable predictions and insights.

Moreover, there are also open technical questions and challenges, for instance regarding sampling a sufficiently large space of possible neuronal models and the related necessity of generating sufficiently large numbers of systematically randomized ‘null’ models of biophysical mechanisms and morphological features.

Many other open scientific questions remain to be addressed. For example, if neurons are optimized for the best compromise between function and energy efficiency, what happens if they face perturbations such as scarcity of energy resources? Interestingly, a recent study [113] has shown that in animals with food restriction, layer 2/3 pyramidal cells (L2/3 PCs) in visual cortex increase their energy efficiency (by weakening their input synapses) but reduce their coding precision (as reflected in a broader orientation tuning). However, the firing rate of L2/3 PCs remained unchanged. It would be intriguing to apply Pareto theory to these data. It is tempting to speculate that under food scarcity, neocortical L2/3 PCs moved along the Pareto front closer towards the archetype for maximum energy efficiency but further away from the archetype for the best computational function in the form of visual information processing. Curiously, they still performed well in firing rate homeostasis. Thus, the neurons probably found a new optimal balance between economy and visual computation. It would be interesting to use population modelling and ion channel analyses to find out the shape of the Pareto front in parameter space of L2/3 neurons. It might be a line for a trade-off between economy-visual processing. Alternatively, it might be a triangle if relevant trade-offs include firing rate homeostasis. Or it could be a tetrahedron or another polytope if these cells are optimized for multi-objective trade-offs between more than three tasks.

Another exciting and not fully resolved question is whether multi-objective Pareto optimality may provide insights on well-established correlations of ion channels. Ion channel correlations have been observed in experiments [114–119] and explored in computational models [30,120,123]. Our hypothesis is that multi-task Pareto optimization of ion channel parameters could shape their homeostatic tuning and lead to ion channel correlations. This is in line for example with the above-mentioned computational prediction that low calcium and potassium channel co-expression appears to be optimal for the trade-off between dendritic computing and low energy cost [91]. Likewise, ion channel expression data from fast-spiking neurons in the vestibular nucleus suggest that co-regulation of ion channels supports optimal balance between high firing rates and their energy efficiency [119]. Other recent computational work using biophysically simple single-compartment models has explored as to when homeostatic co-regulation of ion channels leads to ion channel correlations [124]. Intriguingly, Yang *et al*. [124] have addressed this question in the context of ion channel degeneracy and multi-objective optimization. The authors predict that homeostatic ion channel co-regulation can lead to many (degenerate) multi-objective solutions if the number of available ion channels is higher than the number of objectives. For example, more than two ion channels would be required for finding multiple neuronal models with a successful co-regulation of firing rate and energy efficiency. Thus, homeostatic co-tuning of multiple tasks seems possible only with sufficiently large ion channel diversity (see also [36]). In addition, ion channel correlations seem to arise if the solution space (defined as a difference between the number of dimensions of parameter space and the number of dimensions of performance space) is low-dimensional [124]. It would be interesting to compare these predictions to experiments accompanied by simulations in biophysically and morphologically more complex models complemented by Pareto analysis. Pareto theory suggests that irrespective of the dimensionality of parameter space, the Pareto front for *n* tasks (objectives) is an (*n* – 1)-dimensional surface in parameter space [44]. To add a remark of caution, homeostatic regulation (for instance of firing rate) may generate linear relationships between ion conductances, which might be difficult to distinguish from the correlations resulting from multi-objective optimization (cf. [125]).

## 8. Conclusion

We have seen that Pareto multi-objective optimality is a useful concept for an elegant simplification of the geometry of parameter space. It has been widely employed in engineering, computer science and economics [126–129]. However, only relatively recently has it started being used in molecular biology and in other life science areas [39] including neuroscience [34]. Importantly, it has been successfully applied not only to phenotypes of organisms [102,130] but also to phenotypes of molecules, molecular pathways [38,131–133] and cells, including intestinal and liver cells [100], cancer cells [90] and nerve cells [5,65,71,72].

Therefore, based on these and other examples mentioned here, we believe that Pareto optimality can be fruitfully applied to conductance-based population models of neurons (and their circuits, see [34]), especially if it informs the search for models with optimal trade-offs between economy and neuronal computations and takes advantage of simplifying geometrical rules for Pareto fronts in parameter space [38,44]. In short, we encourage a more frequent usage of Pareto theory and evolutionary economy–effectiveness trade-offs to select optimal and therefore presumably the most realistic neuronal models. Pareto optimality could provide a general conceptual framework to elucidate the diversity in ion channel properties of neurons. This theoretical framework is linked to multi-task evolution theory [38,39] implying that trade-offs between tasks curb ion channel expression to a continuous Pareto front having a shape of a polytope whose vertices represent ion channel expression profiles specializing for a given task (cf. [90]).

## Data Availability

This article has no additional data.
